# Genetic Diversity and Population Structure of a Wide *Pisum* spp. Core Collection

**DOI:** 10.3390/ijms24032470

**Published:** 2023-01-27

**Authors:** Nicolas Rispail, Osman Zakaria Wohor, Salvador Osuna-Caballero, Eleonora Barilli, Diego Rubiales

**Affiliations:** 1 Instituto de Agricultura Sostenible, CSIC, Avda. Menéndez Pidal s/n, 14004 Córdoba, Spain; 2 Savanna Agriculture Research Institute, CSIR, Nyankpala, Tamale P.O. Box TL52, Ghana

**Keywords:** core collection, DArTSeq, genetic diversity, germplasm, peas, *Pisum* spp., population structure

## Abstract

Peas (*Pisum sativum*) are the fourth most cultivated pulses worldwide and a critical source of protein in animal feed and human food. Developing pea core collections improves our understanding of pea evolution and may ease the exploitation of their genetic diversity in breeding programs. We carefully selected a highly diverse pea core collection of 325 accessions and established their genetic diversity and population structure. DArTSeq genotyping provided 35,790 polymorphic DArTseq markers, of which 24,279 were SilicoDArT and 11,511 SNP markers. More than 90% of these markers mapped onto the pea reference genome, with an average of 2787 SilicoDArT and 1644 SNP markers per chromosome, and an average LD_50_ distance of 0.48 and 1.38 Mbp, respectively. The pea core collection clustered in three or six subpopulations depending on the pea subspecies. Many admixed accessions were also detected, confirming the frequent genetic exchange between populations. Our results support the classification of *Pisum* genus into two species, *P. fulvum* and *P. sativum* (including subsp. *sativum*, *arvense*, *elatius*, *humile*, *jomardii* and *abyssinicum*). In addition, the study showed that wild alleles were incorporated into the cultivated pea through the intermediate *P. sativum* subsp. *jomardii* and *P. sativum* subsp. *arvense* during pea domestication, which have important implications for breeding programs. The high genetic diversity found in the collection and the high marker coverage are also expected to improve trait discovery and the efficient implementation of advanced breeding approaches.

## 1. Introduction

Peas (*Pisum sativum* L.) are a multipurpose and low-cost source of protein, that have been an essential source of animal feed and human food for centuries [1]. It is the world’s fourth most cultivated pulse crop [2]. Their usage differentiates cultivated peas into dry peas, green peas and forage peas [3]. The most cultivated are dry peas, traditionally intended for animal feed, but increasingly becoming human food. North America dominates dry-pea production, followed by Europe, while the Asia-Pacific dominates green-pea production (https://www.fao.org/faostat/en/#home, accessed on 24 October 2022). Peas also improve the soil by fixing atmospheric nitrogen through symbiotic interactions with soilborne bacteria. These interactions have a beneficial impact on soil fertility and the nitrogen cycle [4]. Therefore, peas have an excellent potential to improve the livelihoods and protein requirements in regions with low–average protein supply per capita and day. However, pea yield is still unstable due to its limited adaptability to various environmental conditions and susceptibility to diseases and pests. Therefore, great efforts are needed to improve its adaptation and resistance to biotic and abiotic stresses [1,5].

Peas are among the world’s oldest domesticated crops, with evidence of domestication dating back 10,000 years. The crop centre of origin and diversity is the Near East, with secondary diversification regions in the Mediterranean and East Africa. *Pisum* is a member of the family *Fabaceae*, the subfamily *Papillionaceae*, and the tribe *Vicieae*. The taxonomy of the genus *Pisum* is very complex and still under debate, with some authors recommending its inclusion in the genus *Lathyrus* [6]. It is generally accepted that *Pisum* includes three species: *P*. *sativum*, *P*. *fulvum* and *P*. *abyssinicum* [7,8,9]. However, the status of *P*. *abyssinicum* as an independent species, or as a *P*. *sativum* subspecies, is still controversial [10]. *P*. *sativum* is the primary and more diverse species of the genus. This species has been recently separated into two subspecies: subsp. *sativum* that contains cultivated peas and subsp. *elatius* that contains wild peas [11]. Each *P*. *sativum* subspecies is further divided into varieties, including the cultivated var. *sativum* and var. *arvense*, and the wild var. *elatius* and var. *pumilio* [7], a synonym of *P*. *sativum* var. *humile* and *P*. *sativum* var. *syriacum* [12,13]. Additional wild subspecies have also been described, including subsp. *jomardii* [14], subsp. *thebaicum*, subsp. *transcaucasicum*, subsp. *asiaticum* and subsp. *cinereum,* although their taxonomy status remains unclear.

All *Pisum* species and subspecies are crossable, albeit fertile hybrids between wild and cultivated peas may be obtained at a low rate [15]. Breeding programs may benefit from this wealth of natural diversity. Many *ex situ* pea germplasm collections have been developed to provide long-term conservation and ready access to a broad range of diversity [8]. These collections include wild, local landraces, commercial varieties and mutants. They are rich in genetic diversity for many traits of agronomical interest, including growth habits, seed quality and resistance to stress [16]. Several core collections have been developed to facilitate pea breeding programs [3,8,17], that significantly improve pea yield and quality through classical breeding [3,16].

Several approaches have been applied in pea breeding programs, including bulk selection, pedigree breeding schemes through transgressive segregation, single-seed descent and backcross selection, for scouting single dominant traits [2]. More recently, molecular marker technology has introduced new dimensions for improving traits of interest [2]. This technology facilitates genetic diversity studies to provide essential information for genetic conservation and efficient breeding of new commercial varieties [2,18]. They also allow for the deployment of linkage maps that localize specific genetic regions in the genome, and identify flanking markers associated with valuable traits [18]. Extensive genetic maps have been established based on bi-parental mapping populations combining different markers, including morphological markers, isozymes, RFLPs, RAPDs, SSRs and SNPs [19]. The knowledge collected from these markers led to the development of a consensus composite map of 1430 cM, comprising 239 microsatellite markers helpful in locating QTLs controlling disease resistance, as well as quality and morphological traits [19].

More recently, advances in sequencing technology opened the possibility of implementing genome-wide association studies (GWAS) and genomic selection (GS) to facilitate and boost future QTL identification and breeding [2]. The efficient implementation of these advanced breeding approaches depends on the molecular markers’ genome coverage, the extent of linkage disequilibrium (LD) between these markers and the population structure of the germplasm collection [20,21]. In addition, germplasm collections should contain high genetic diversity with a wide variation for the traits of interest. A detailed description of genetic diversity and population structure is a crucial prerequisite to their implementation [20]. Furthermore, genetic diversity is a significant determinant of a species’ capacity to persist and adapt to their environment. Unravelling genetic differentiation factors may explain how species react to changing environments [22]. Many studies aimed to clarify the genetic diversity of peas with diverse molecular markers and germplasm core collections [2]. The results of these studies were highly variable depending on the composition of the collection and the method employed. However, they all demonstrated a high genetic richness within peas and highlighted the complex population structure that explains the unresolved pea taxonomy [23,24].

In this work, we established the population structure and genetic diversity of a pea core collection containing 325 accessions from a worldwide origin. The collection includes all *Pisum* species and subspecies, to shed further light on the pea phylogenetic relationship, and serve as a first step toward implementing GWAS and GS in peas for agronomic traits and disease resistance.

## 2. Results

### 2.1. The Pea Core Collection

**Figure 1:**
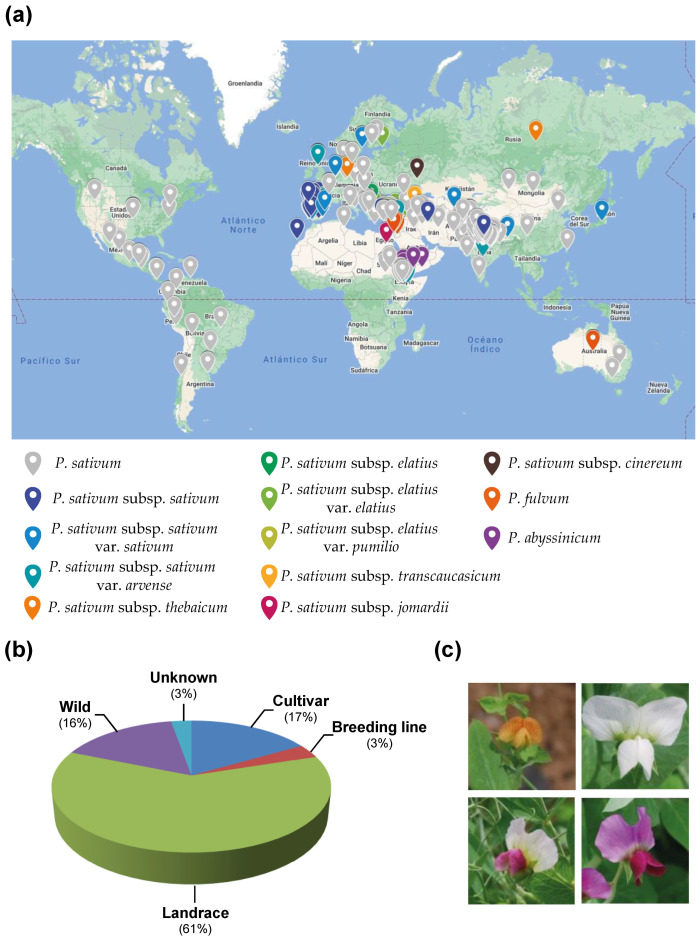
The IAS pea core collection. (**a**) Geographic distribution of the pea accessions that compose the IAS pea core collection based on Global Positioning System (GPS) data from available passport data. Different colours differentiate pea species or subspecies. (**b**) Distribution of the IAS pea core collection based on the plant material type (wild, landraces or cultivars). (**c**) Examples of the different types of flowers observed within the IAS pea core collection.

The Instituto de Agricultura Sostenible (IAS) pea core collection contained 325 accessions. Pea accessions of this core collection were selected based on geographical and morphological diversity to preserve the underlying levels of genetic diversity. Disease resistance was at the base of the germplasm-gathering process. Therefore, previously described sources of disease resistance including powdery mildew, rust, ascochyta blight, fusarium wilt and broomrape, were included in the core collection [25,26,27,28,29]. Although some commercial varieties and breeding lines were included, the predominant accessions were landraces, representing 61% of the collection. Wild species represented 16% of the collection. The collection was further selected based on flower-colour variability and contained accessions with white, purple, pink, lilac and orange flowers (Figure 1, Table 1 and Supplementary Table S1). Preliminary evaluation of this collection to rust showed a wide variety of responses, from susceptible to resistant accessions, indicating its suitability for further genetic study of disease resistance [30].

### 2.2. DArTSeq Marker Sequencing and Genetic Diversity Indices

DArTSeq sequencing and assembling of the collection led to the identification of 66,643 SilicoDArT and 55,269 SNP markers. After the stringent data curation according to minor allele frequency (MAF), heterozygosity and missing values, 24,279 polymorphic SilicoDArT markers and 11,511 SNP markers were obtained. Mapping these DArTSeq markers onto the pea reference genome located 19,514 SilicoDArT and 10,125 SNP markers on the seven pea chromosomes (82.8% of the DArTSeq markers). In addition, 2703 SilicoDArT and 1155 SNP mapped to unanchored pea contigs and supercontigs (10.8% of the DArTSeq markers), while 2062 SilicoDArT and 231 SNP markers remained unaligned (6.4% of the DArTseq markers). Sequence read data from these accessions are available at the NCBI SRA archive as BioProject PRJNA890072. The DArTSeq marker datasets were deposited in the public Zenodo depository [31].

To estimate the genetic diversity of the IAS pea core collection, we estimated the polymorphic information content (PIC), MAF, allele richness (Ar), expected heterozygosity (H_e_), observed heterozygosity (H_o_) and inbreeding coefficient (F_IS_) for each DArTSeq marker (Table 2). Wide variability for all parameters evaluated was observed across markers. For SilicoDArT, PIC values varied from 0.006 to 0.499, with an average value of 0.295, while MAF varied from 0.052 to 0.997, with a mean value of 0.41. H_e_ values were slightly lower than PIC, varying from 0 to 0.5, with a mean value of 0.217. Wide variation was also observed for the inbreeding coefficient that varied from −0.102 to 1, with a mean value of 0.692. Results obtained for SNP markers were broadly similar to those estimated for SilicoDArT markers (Table 2).

### 2.3. Marker Distribution and Linkage Disequilibrium

DArTSeq marker distribution over the pea genome showed a homogeneous distribution, covering the full length of the seven chromosomes (Supplementary Figure S1 and Table 3). Although the coverage of both marker types was similar, the density of SilicoDArT markers doubled that of SNP markers (Table 3). The average number and distance between SilicoDarT markers were *circa* two-fold that of SNP markers, reaching 2787.7 markers per chromosome, separated by 0.166 Mbp for SilicoDArT markers and 1446.4 markers, separated by 0.319 Mbp for SNP markers, respectively (Table 3). Slight variations were also detected in the marker distribution between chromosomes, with chromosome 2 presenting the lowest values and chromosome 5 the highest (Table 3). Interestingly, the middle region of chromosome 2 was inferior in molecular markers.

**Figure 2:**
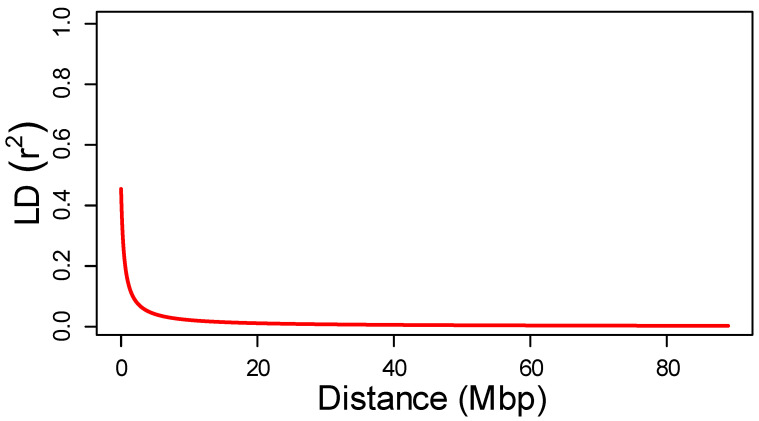
Estimation of the average distance of LD decay. LD decay plot showing pairwise LD values (r^2^) on the *x*-axes plotted against genetic distance Mbp on the *y*-axes. The fitted red line is a nonlinear log curve of r^2^ on genetic distance.

Inspection of intra-chromosomal linkage disequilibrium (LD) indicated that r^2^ varied from 0.012 to 1 for both SilicoDArT and SNP markers. The mean LD value and the overall critical value of LD (r^2^_90_), estimated as the ninetieth percentiles of r^2^, were also similar between marker types reaching values of 0.103 and 0.236 for SilicoDArT, and 0.118 and 0.28 for SNP markers, respectively (Table 4). Plotting the r^2^ value over the physical distance between markers shows the rapid LD decay reaching LD_50_, at 0.48 and 1.38 Mbp for SilicoDArT and SNP markers, respectively (Figure 2). Estimation of LD by chromosomes shows slight differences in the extent of marker linkage between chromosomes. Chromosome 6 showed the highest LD_50_, mean r^2^ and r^2^_90_ values, while chromosome 7 showed the lowest (Table 4, Supplementary Figure S2).

### 2.4. Genetic Structure of the Pea Core Collection

**Figure 3:**
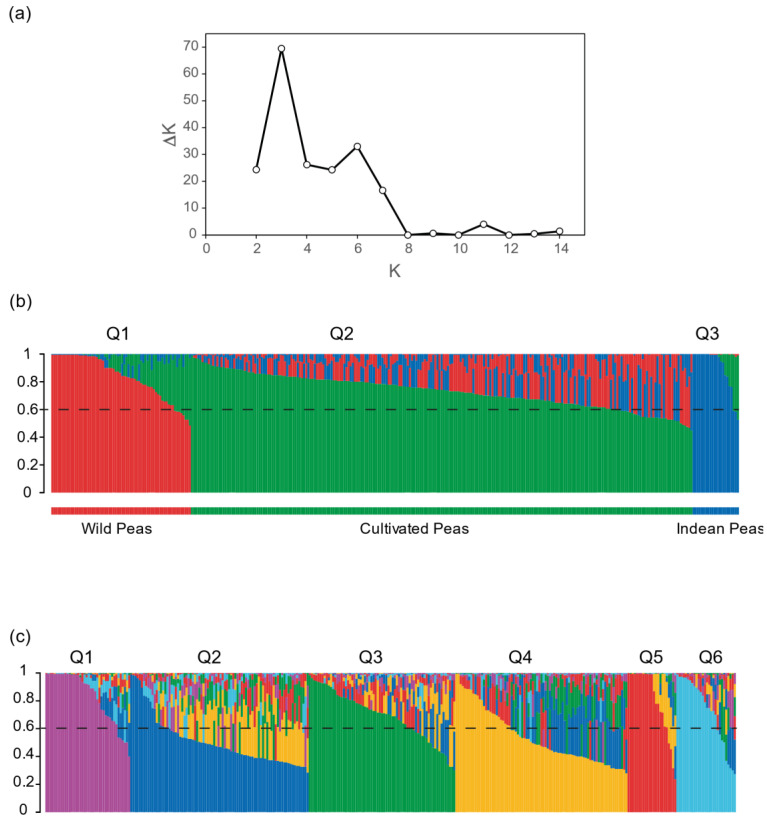
Population structure of the IAS pea core collection as estimated by STRUCTURE. (**a**) Estimating the optimum subpopulation number was based on Evanno’s parameter (ΔK). (**b**) STRUCTURE output for K = 3. (**c**) STRUCTURE output for K = 6. Each bar of the histograms represents the percentage of membership to each STRUCTURE subpopulation of a given pea accession from the IAS pea core collection.

The genetic structure of the pea core collection was analysed with the model-based software STRUCTURE after LD pruning of the SilicoDarT marker matrix, as this database showed the highest chromosomal coverage. The evolution of the Evanno parameter (Δk) showed two peaks, suggesting the presence of three or six subpopulations (Figure 3a). According to STRUCTURE output for K = 3 (Figure 3b and Table 5), the first group (Q1), containing 28.16% of the collection, was constituted by wild pea relatives, including all accession of *P. fulvum*, *P.* abyssinicum, *P. sativum* subsp. *elatius* var. *pumilio*, *P. sativum* subsp. *transcaucasicum* and most *P. sativum* subsp. *elatius* var. *elatius* accessions. The second group (Q2), representing 56.83% of the collection, contained the cultivars and landraces of *P. sativum* subsp. *sativum* var. *sativum*, *P. sativum* subsp. *sativum* var. *arvense*, *P. sativum* subsp. *jomardii*, *P. sativum* subsp. *thebaicum* and *P. sativum* subsp. *cinereum*. This group included six *P. sativum* subsp. *elatius* var. *elatius* accessions; two had a shared membership percentage (*circa* 50% Q2 and 45% Q1). The third group (Q3), containing 15.01% of the collection, was composed of a smaller set of *P. sativum* landraces from India (Figure 3b and Table 5).

For K = 6, the group of wild accessions was further divided into two, representing 14.25 and 9.37% of the collection, respectively (Figure 3c and Table 4). The first sub-group (Q1) contained the *P. fulvum* and *P. abyssinicum* accessions and most of the *P. sativum* subsp. *elatius* var. *elatius.* By contrast, the second and smallest wild sub-group (Q6) contained the *P. sativum* subsp. *elatius* var. *pumilio* accessions [11]. At K = 6, the group of the cultivated subspecies was also separated into three sub-groups, each representing 21% of the collection (Table 5). The first one (Q2) contained landraces of *P. sativum*, all accessions from the *P. sativum* subsp. *jomardii* and *thebaicum*, and two accessions initially assigned to *P. sativum* subsp. *elatius.* The second sub-group (Q3) contained the *P. sativum* subsp. *sativum* var. *sativum* cultivars, while the last sub-group (Q4) contained all the landraces from *P. sativum* subsp. *sativum* var. *arvense.* Finally, the Indian *P. sativum* landraces, that represented 12% of the collection, composed the Q5 sub-group (Figure 3c).

**Figure 4:**
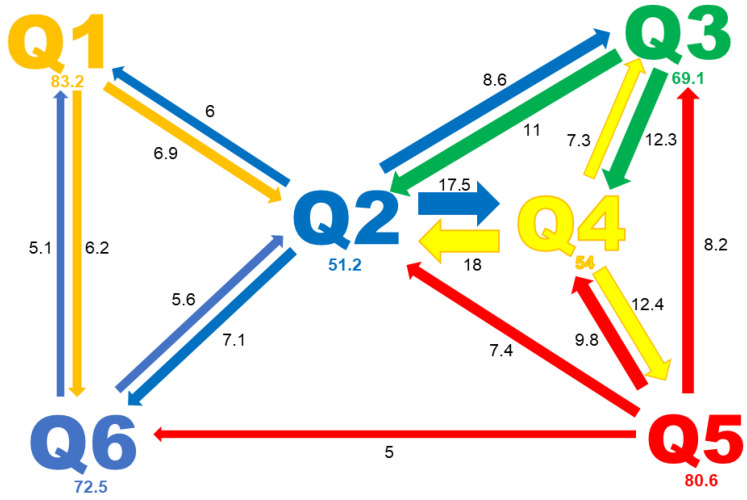
The relationship between the different subpopulations of the IAS pea core collection. The relations between subpopulations were based on the genetic proportion shared between them, as estimated by STRUCTURE. The arrow indicates the donor and receiver subpopulations. The numbers indicate the mean proportion of membership (in %) given by the donor subpopulation. Only contributions of at least 5% are presented.

This analysis also revealed a high level of admixture, with 46 and 155 accessions showing a percentage of membership to any subpopulation lower than 60% for K = 3 and 6, respectively. Most accessions with less than 60% of membership to a given group were assigned to Q2 and Q4, which represented 78.6% and 68% of admixed accessions, respectively. The proportion of admixed accessions for the other groups was lower than 30%. The mean membership percentage was 51.2% and 54% for Q2 and Q4 (Figure 4), approximately 70% for Q3 and Q6, and above 80% for Q1 and Q5. This analysis also revealed that members of the Q2 subpopulation shared genetic information with all other subpopulations. This subpopulation of domesticated peas shares approximately 6% of genetic information with each of the wild subpopulations (Q1 and Q6). None of the other domesticated pea subpopulations showed evidence of sharing significant genetic information with these wild subpopulations. By contrast, Q2 and Q4 shared approximately 18% of genetic information from each other. This analysis also revealed an important shared history between Q4, Q3 and Q5 subpopulations (Figure 4).

Estimation of the fixation index (F_st_) for each subpopulation pointed to the significant divergence between subpopulations (Table 5). Estimated F_st_ values ranged from 0.2268 to 0.4315 for K = 3, and from 0.3316 to 0.5404 for K = 6, respectively (Table 5). The genetic distance between accessions within each subpopulation varied from 0.1514 to 0.2262 for K = 3, and from 0.1305 to 0.2141 for K = 6 (Table 5).

### 2.5. Principal Component Analysis

**Figure 5:**
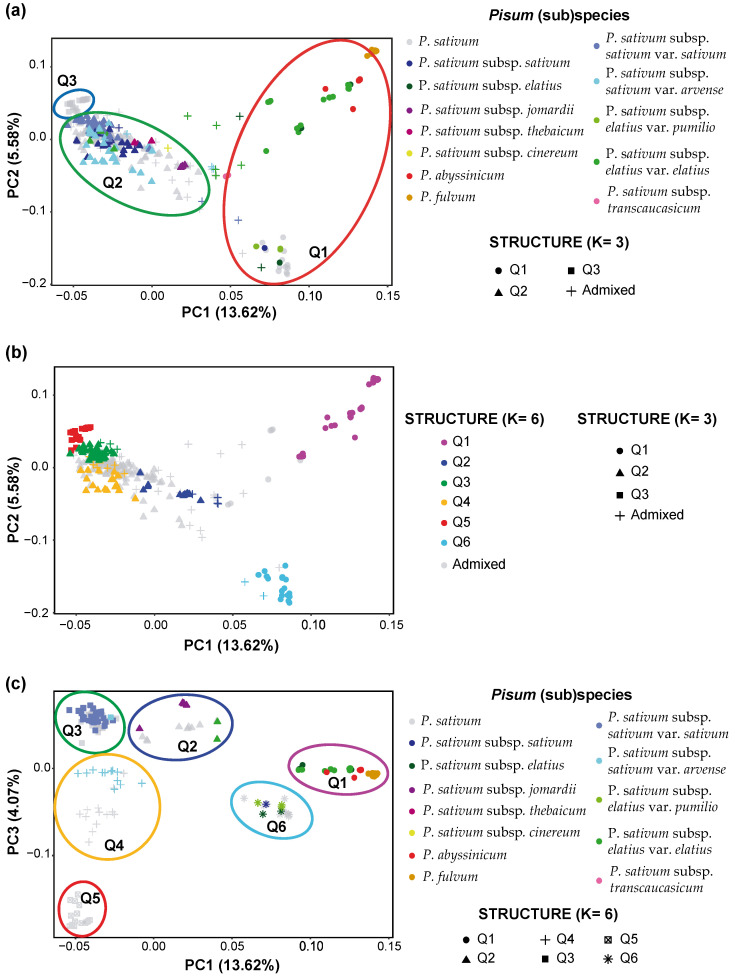
Population structure of the IAS pea core collection as estimated by PCA. The figure shows the PCA analysis of the 11,635 polymorphic SilicoDArT markers for the 325 accessions of the IAS pea core collection. (**a**) Scatterplot of the first two PCs. Each accession is represented by a coloured symbol depending on its STRUCTURE subpopulation for K = 3 and its pea subspecies. (**b**) Scatterplot of the first two PCs with information of the STRUCTURE output for K = 6. (**c**) Scatterplot of PC1 *vs*. PC3 that allow a better separation of the six STRUCTURE subpopulation. Each accession is represented by a coloured symbol depending on its STRUCTURE subpopulation and subspecies.

To clarify the genetic structure of the pea core collection, principal component analysis (PCA) was performed to highlight the different variables that could explain the splitting of this population. The initial three principal components (PCs) explained 23.3% of the observed genetic variation in the pea collection. The first PC explained up to 13.62% of the variation, the second explained 5.58 and third PCs explained 4.07%. The first two PCs clustered the pea collection in three different groups (Figure 5a,b). The first group included *P*. *fulvum*, *P*. *sativum* subsp. *elatius* var. *elatius* and *P*. *abyssinicum* accessions. The second group contained the *P*. *sativum* subsp. elatius var. *pumilio*. The last, and most significant group, contained domesticated *P. sativum*.

Superposing the STRUCTURE clustering for K = 3 or K = 6 to the PCA clustering favours the estimation of six subpopulations (Figure 5b). In this case, the two smaller PCA groups corresponded to the STRUCTURE subpopulation Q1 and Q6 respectively. The other STRUCTURE subpopulations were included within the leading PCA group in four distinct clusters (Figure 5b). The presence of six subpopulations was further supported after plotting PC1 *vs* PC3 which revealed six clusters, corresponding to the six subpopulations previously estimated by STRUCTURE for K = 6 (Figure 5c).

### 2.6. Phylogenetic Relationship of the Pisum Core Collection

**Figure 6:**
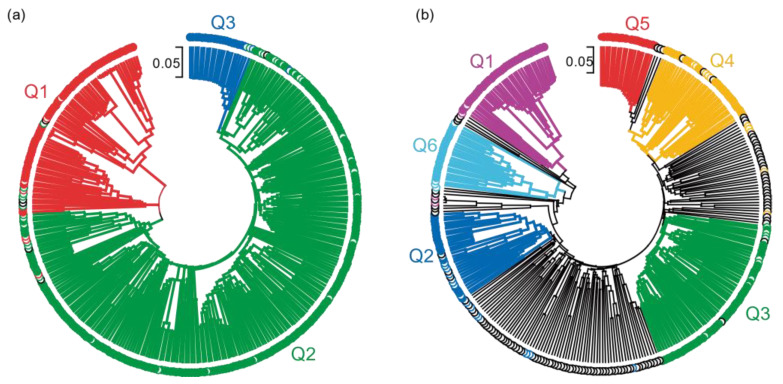
Phylogenetic relationship of the IAS pea core collection. The phylogenetic relationships were inferred using the Neighbor-Joining method with 1000 bootstraps. The tree was drawn to scale, with branch lengths corresponding to the number of differences per site. The evolutionary distances were computed using the p-distance method. The rate variation among sites was modelled with a gamma distribution (shape parameter = 0.98). All positions with more than 5% missing data were eliminated (partial deletion option). There was a total of 11,635 markers in the final dataset. The branch of each accession was coloured depending on its STRUCTURE subpopulation (Q) for K = 3 (**a**) or K = 6 (**b**).

The Neigbor-Joining (NJ) tree resolved into six distinct groups, as shown in Figure 6. Comparing the NJ tree with the STRUCTURE and PCA results revealed considerable congruence. For K = 3, subpopulation 1 was formed by two distinct clusters located at the base of the phylogenetic tree. Subpopulation 2 was composed of four distinct clusters. Subpopulation 3 appeared as a small cluster closely related to subpopulation 2, further supporting the PCA results (Figure 6a). The congruence between PCA, STRUCTURE and the phylogenetic analysis was more robust for K = 6 where each STRUCTURE subpopulation corresponded to distinct clusters on the NJ phylogenetic tree (Figure 6b). Interestingly, each subpopulation was separated by admixed accessions (% membership < 60%), supporting a significant history of hybridization and mixture between populations along evolution. Similarly, subpopulation 2 clustered between wild and domesticated pea subpopulations, while Q4 was located between Q3 and Q5.

## 3. Discussion

Broadening crop genetic diversity is critical for efficient breeding. Therefore, the conservation and characterisation of crop genetic resources is a crucial breeding element. The development of core collections representative of the crop genetic diversity allows the successful exploitation of genetic diversity richness, and protects against genetic erosion. These core collections, therefore, represent unique genetic diversity donors to enhance genetic gain, boost production and reduce stress-induced losses [17,32,33]. The constant cost decrease and higher efficiency of crop genotyping significantly increase the opportunity to characterise and exploit these collections. Although phenotypic evaluation is still a prerequisite to their efficient exploitation, incorporating genetic maps with high-density marker coverage and efficient bioinformatic tools can optimize their evaluation and usefulness [32,33].

Despite pea agronomic importance and long cultivation history, its domestication events and population dynamics still need to be better understood [22,24]. The development of diverse pea core collections and their detailed characterisation at genetic and population-genomic levels are improving our understanding of pea evolutions, and may ease their wide exploitation in breeding programs through the implementation of quantitative genomic approaches (GWAS and GS) [8,17,32]. The large size and complexity of the pea genome have largely delayed genomic research on this crop compared to other crops. However, the recent development of NGS-based genotyping facilitates the development of thousands of genome-wide molecular markers. In addition, the release of the pea reference genome sequences increased the scientific community’s interest, rapidly closing the gap, providing new insights into pea domestication and boosting pea breeding [2,9,17,34]. Accordingly, the carefully selected pea-core collection was extensively analysed for genetic diversity and population structure (Figure 1). This collection was designed to widen the available pea genetic diversity and exploit disease resistance traits. Therefore, the previously-described source of (partial) resistance to the most prevalent pea diseases was included in the collection. This collection partially overlaps with previously-developed pea panels designed to untangle pea domestication history [24,35] or contribute to pea breeding [17,20]. Eighty accessions (24.6% of the collection) are shared in at least one previously-described pea panel, which allows comparing and integrating the results of the different studies to get further insight into the pea genetic diversity and population dynamics.

DarTSeq genotyping approach has emerged as a proper genomic method for GS, genetic mapping and population genetics approaches in many plant species [36,37]. Application of this GBS-related approach in the present study provided a high-density coverage of the pea genome and yielded reliable data. It allowed for the generation of 35,790 polymorphic DArT markers, of which 24,279 were SilicoDArT and 11,511 SNP markers. These results were similar to previous studies on a pea panel genotyped with the DArTSeq approach that identified 35,647 DarT markers [24] and 11,343 SNP markers [35]. It was also similar to the number of SNP markers obtained from pea panels with the widely used Genopea Infinium SNP array [23,38,39], and in the same order of magnitude as the GBS approach [32,40]. In addition, estimation of the genetic diversity DArTSeq markers harbour moderate level of genetic diversity, with PIC and H_e_ mean values of 0.295 and 0.217, respectively. However, the genetic diversity harboured by each marker varied largely (Table 2), which is similar to previous studies using DArTSeq technology [41,42]. As such, our results confirm the capacity of DArTSeq to provide high-throughput genome-wide polymorphism markers.

Interestingly, the total number of DArTSeq markers collected here was three-fold higher than a previous DArT sequencing of peas based on several RIL populations [37]. The ratio between SilicoDArT marker and SNP markers was broadly similar between both studies. This difference in the total number of polymorphic markers between diverse pea collections and RILs was in accordance with previous results, confirming the lower RIL population genetic variability [40,43].

More than 90% of the DArT markers could be mapped onto the seven pea chromosomes and unaligned supercontigs. The alignment of DArTSeq markers onto the reference pea genome showed good coverage of all pea chromosomes. We observed an average of 2787 SilicoDArT and 1644 SNP markers per chromosome, evenly distributed across the chromosomes, which is in accordance with previous studies on peas (Table 3) [38]. A high number and genome-wide distribution of molecular markers are paramount for subsequent quantitative genomic approaches, such as GWAS and GS. The high number of markers and their broadly-even distribution onto the pea chromosomes, with the polymorphic markers detected here, should allow the successful implementation of GWAS and GS approach with this pea core collection.

Population structure and LD are the main obstacles to identifying significantly-associated markers with phenotypic traits [44]. The power of association studies depends on the existing LD between the gene(s) controlling phenotypic trait(s) and associated marker(s). Our data showed an extensive LD between markers. The LD-decay estimates on the seven pea chromosomes varied from 0.24 to 1.05 Mbp (mean 0.48 Mbp; Figure 2 and Table 4), which represented approximatively from 0.04 to 0.19 cM (mean 0.09 cM), agreeing with previously-estimated correspondence of 1 cM per 5.6 kb [23]. A recent study obtained after the pea reference genome release indicated a significantly smaller distance of LD decay, varying from 0.03 to 0.18 Mbp [40]. However, this difference in the LD-decay distance may be due to differences in the method used to estimate the LD_50_ distance. In the present study, LD_50_ distance corresponded to the physical distance in Mb at which LD had decayed to half of the r^2^ max. At the same time, it was limited to the r^2^_max,90_ in the previous study, because the r^2^_max,90_ for each chromosome was similar between both studies (Table 4) [40]. Additionally, our LD_50_ estimation was in the same order of magnitude as previous studies that estimated an LD_50_ distance between 0.05 to 0.9 cM, depending on the pea panel [23,38,39]. The rapid LD decay and the high chromosomal coverage, with markers at an average of 0.166 Mbp within the LD-decay window, should ensure the efficient and precise delineation of QTLs in future GWAS studies.

Population structure analysis of the IAS pea core panel through PCA, STRUCTURE and phylogenetic approaches differentiated three or six subpopulations (Figure 3, Figure 5 and Figure 6). This study also uncovered many admixtures with nearly half of the pea accessions having less than 60% membership to any subpopulation for K = 6. These results confirm the reproductive compatibility existing between *Pisum* species and subspecies [11]. They also indicated that even if self-pollination is the predominant mode of reproduction and some reproductive barriers exist, hybridization between accessions from different *Pisum* species and subspecies frequently occurred during pea evolution and domestication [11,24,35,45]. The Evanno’s method used to determine the number of pea populations from STRUCTURE revealed a major peak of Δk for K = 3 and a secondary peak at K = 6 (Figure 3). This indicated that the pea core panel was composed of three main populations (Figure 3). The wild pea accessions, at K = 6, included the species *P*. *fulvum* and *P*. *abyssinicum*, and the wild *P*. *sativum* subsp. *elatius* clustered together in one group (Q1). Cultivated *P*. *sativum*, including *P*. *sativum* subsp. *sativum* var. *sativum* and *P*. *sativum* subsp. *sativum* var. *arvense* clustered in two additional groups (Q2 and Q3). The Q2 clustered most of the cultivated *P*. *sativum* accessions, and the Q3 were pea landraces from the northern regions of India. This clustering was supported by the phylogenetic analysis, while the PCA results separated the wild population into two distinct groups, suggesting additional genetics clusters (Figure 5). The lower number of populations estimated by STRUCTURE may be due to the approximation used by the Evanno’s method that often underestimates the number of genetic clusters [46,47,48,49].

Accordingly, the three main groups of the pea core panel can be further differentiated into six clusters separated by the NJ trees and the PC1 vs. PC3 representation of the PCA analysis. In this latest analysis, PC1 discriminated accessions based on their domestication degree (wild or domesticated), while PC3 discriminated among the subspecies. In this grouping, the wild group was separated into two clusters. The first cluster contained the *P*. *fulvum, P*. *abyssinicum* and most accessions of *P*. *sativum* subsp. *elatius* var. *elatius* (Q1). The second included all accessions assigned to *P. sativum* subsp. *elatius* var. *pumilio* (Q6). The division of these two varieties confirmed their genetic distinctiveness, as previously shown by examining several wild populations from Israel and other Mediterranean regions that differentiated between *P*. *sativum* subsp. *elatius* var. *elatius* and *P*. *sativum* subsp. *elatius* var. *humile* [22,50].

On the other hand, the domesticated groups formed four distinct clusters. This result confirmed the genetic distinctiveness of the subset of Indian *P. sativum* accessions that formed the Q5 cluster. Central Asia (covering the highland Asiatic region from Afghanistan, the Hindu Kush and along the length of the southern slopes of the Himalayan mountains, as well as the central areas of China) is a relevant secondary centre of pea diversity that is thought to be rich in primitive cultivated forms of field peas [51,52]. Previous population genomics studies on these regions demonstrated a greater diversity within these regions than worldwide [51], providing evidence for the existence of separate gene pools from northcentral China and Afghanistan [13,52]. A similar situation might exist in Indian northern regions, given the geographical proximity, leading to this separated pea gene pool. The overlapping of the pea panels used for these different studies hamper the comparison of the genetic diversity held between these different central-Asian gene pools. Therefore, further studies targeting central Asia would be needed to clarify the relation between these gene pools and reconstruct their domestication history.

The rest of the domesticated accessions were divided into three clusters; one containing all the *P*. *sativum* subsp. *sativum* var. *sativum* cultivars and landraces (Q3), and two large groups of highly admixed accessions containing the *P*. *sativum* subsp. *jomardii* (Q2) and *P*. *sativum* subsp. *sativum* var. *arvense* (Q4). Most accessions assigned to Q2 and Q4 by STRUCTURE showed a membership coefficient lower than 60%; 78.6% of the Q2 accessions and 68% of the Q4 accessions were admixed. The percentage of admixed accessions from other clusters only varied from 15 to 30%. Further examination of the individual membership coefficient from each cluster indicated many hybridization events between Q2 accessions and the wild subpopulations (Q1 and Q6). The results revealed the existence of a frequent genetic exchange between Q2 and Q4, and between Q4 and the other two domesticated clusters (Q3 and Q5) (Figure 4). This suggests that *P*. *sativum* subsp. *jomardii* and *P*. *sativum* subsp. *sativum* var. *arvense* subpopulations arose during pea domestication, and are intermediate populations between the wild and domesticated genotypes. Therefore, these two subpopulations have a high potential for pea breeding. Our results for the Q2 subpopulation support and illustrate the condition of *P*. *sativum* subsp. *jomardii* as a domestication intermediate, previously proposed by Kosterin and co-workers [14,53].

All previous population genetic analyses separated the wild from domesticated peas and, depending on the scope of the study, further separated the wild or the domesticated pea accessions into several clusters. Depending on the study, pea accessions were grouped based on their species/subspecies [13,22,35], geographic origin [50] and end-use or sowing types [20,38]. The present study separated wild and domesticated pea accessions and separated each group into two and four subpopulations (Figure 3 and Figure 5). While two wild subpopulations were detected, our data could not separate the *P*. *fulvum* and *P*. *abysinicum* from the wild *P*. *sativum* subsp. *elatius* var. *elatius* accessions in contrast with most previous studies [22,35], although all *P*. *fulvum* clustered together in a slightly separated group within Q1 (Figure 3). This might be due to the relatively-low number of accessions belonging to *P*. *fulvum* and *P*. *abysinicum* contained in the IAS pea core collection. For the other subpopulations, pea accessions were separated based on the *P*. *sativum* subspecies, with *P*. *sativum* subsp. *sativum* var. *arvense* forming Q4 and the *P*. *sativum* subsp. *jomardii* and *P*. *sativum* subsp. *thebaicum* forming Q2. Although the overlapping between the IAS pea core collection and the USDA collection was limited, the percentage of accessions from each *Pisum* subspecies was similar between both core collections. Accordingly, the PCA analysis produced identical results (Figure 5a), apart from the *P*. *fulvum* accessions [17]. Similarly, no clustering based on the geographic origin of the accessions could be detected from our data, except for Q5 (Figure 3) [17]. The IAS core collection mostly contained landraces and wild accessions, with limited passport information related to end-uses and sowing types. The available information showed no clear grouping from end-uses or sowing types, although all winter peas clustered in Q2 (Supplementary Table S1).

The present study supports several taxonomic considerations. Firstly, many studies demonstrated that the domesticated *P*. *abyssinicum* is distinct from *P*. *sativum* and arose from an independent domestication event [35,54]. However, the two species shared several agronomy traits, such as pod indehiscence and the lack of seed dormancy [10]. Consequently, Weeden [10] postulated that “if forms of wild ‘*elatius*’, which is largely divergent from *P. sativum* subsp. *sativum,* is not defined at the species level, there is no justification for defining *P*. *abyssinicum* as a species either”. Therefore Weeden [10] proposed its classification as a subspecies within *P*. *sativum.* In our study, the *P*. *abyssinicum* accessions could not be differentiated from the *P*. *sativum* subsp. *elatius* accessions (Figure 3 and Figure 5). This result suggests that the genetic differences between the “*abyssinicum*” and “*sativum*” forms and the “*elatius*” and “*sativum*” forms are similar, and agrees with Weeden’s considerations. Several studies targeting the origin of the Abyssinian peas also supports Weeden’s taxonomic classification [35,54], although others favoured its consideration as a species [7,9]. Secondly, the taxonomic classification of the wild *P*. *sativum* is still highly controverted. Most authors followed the Maxted and Ambrose [7] taxonomic classification, as we did in the present study. According to this classification, all wild peas belong to the *elatius* subspecies. Contrarily, other authors classified the wild pea into several wild subspecies, including *P*. *sativum* subsp. *elatius*, *P*. *sativum* subsp. *humile*, *P*. *sativum* subsp. *jomardii* and *P*. *sativum* subsp. *transcaucasicum* [12,14]. The analysis of the IAS core collection separated the wild accessions into two groups, with all *P*. *sativum* subsp. *elatius* var. *pumilio* accessions forming the Q6 cluster (Figure 3 and Figure 5). This clustering pattern demonstrates that these accessions form a genetically-distinct group among wild peas, confirming recent studies on a wild-pea population that separated the “*southern humile*” (syn. *P*. *sativum* subsp. *humile* var. *humile*) from the “*northern humile*” (syn. *P*. *sativum* subsp. *humile* var. *syriacum*) and “*elatius*” accessions [22,50]. These observations favour the differentiation of *P*. *sativum* subsp. *humile* from the *P*. *sativum* subsp. *elatius*. Therefore, these observations support the classification of *P*. *sativum* subsp. *humile* as a subspecies within the *P. sativum* complex, as some authors considered [12,22]. Thirdly, our results showed that all accessions previously defined as *P*. *sativum* subsp. *jomardii* were placed in the Q2 cluster together with many undefined *P*. *sativum* accessions. This result demonstrates that these accessions are genetically distinct from the other subpopulations. Despite *P*. *sativum* subsp. *jomardii* being described in 1818 [55], its taxonomic classification has only been clarified recently as a *P*. *sativum* subspecies based on three genetic loci [14]. Further studies postulated *P*. *sativum* subsp. *jomardii* as an intermediate population between wild and cultivated peas [53]. Our observations supported the assignment of these pea accessions to a distinct *P*. *sativum* subspecies. Our phylogenetic and population genetic structure analyses support that this *P*. *sativum* subsp. *jomardii* forms an intermediate population during pea domestication (Figure 3, Figure 5 and Figure 6). The Q2 subpopulation also contains the three accessions from the uncertain *cinereum* and *thebaicum* subspecies. Given our analyses, these additional subspecies can be proposed as synonyms of *P*. *sativum* subsp. *jomardii*. Fourthly, all *P*. *sativum* subsp. *sativum* var. *arvense* accessions clustered together in subpopulation Q4. In the same logic as for *P*. *sativum* subsp. *jomardii* and *P*. *sativum* subsp. *humile*, these results argue in favour of restoring the *arvense* subpopulation to the rank of subspecies, as formerly recognised [34]. This recognition is supported by previous analysis of an Australian panel with SSR markers that separated the *arvense* accessions into a distinct subpopulation with *P*. *fulvum* and *P*. *abyssinicum* [13]. By contrast, our analysis does not support the taxonomic classification of the *P*. *sativum* subsp. *transcaucasicum* accessions included in the IAS core collection. This subspecies, described from an expedition to the Caucasus region, was already under debate since several previous studies failed to separate them as independent entities [13,14,35]. According to STRUCTURE, these two accessions (accessions 110 and 263) were highly admixed, sharing approximately 30% of their genome information between Q1, Q2 and Q6. Therefore, our data do not support the assignment of these accessions to a distinct *P*. *sativum* subspecies. Our data, rather, identified them as hybrids, potentially the precursor of the Q2 subpopulation. Altogether, our results would support the classification of the pea family into two species (*P*. *fulvum* and *P*. *sativum)* and the subdivision of *P*. *sativum* into at least five subspecies (subsp. *abyssinicum*, subsp. *elatius*, subsp. *humile*, subsp. *jomardii* and subsp. *arvense)*. Clarifying pea taxonomy is an important step towards efficiently exploiting pea germplasm and genetic diversity for breeding.

Altogether, our study confirms the high genetic diversity of peas and their complex population structure. Our data also support the taxonomic subdivision of *Pisum* in two species, and at least five subspecies of *P. sativum.* This work also highlights that, despite the extensive pea domestication history and its primary autogamic reproduction mode, cultivated peas have maintained a very high genetic diversity, valuable for breeding. In particular, population structure analysis showed that wild alleles had been incorporated into the domesticated pea through the intermediate *P*. *sativum* subsp. *jomardii* and *P*. *sativum* subsp. *arvense*, that form two highly-admixed subpopulations. The high genetic diversity in the IAS pea core collection and the high genome coverage with polymorphic markers also allow the efficient implementation of GWAS and GS approaches. These techniques will be very valuable in improving resistance to major pea diseases through breeding.

## 4. Materials and Methods

### 4.1. Plant Material

The pea core collection used in this study consisted of 325 accessions carefully selected from a large *Pisum* spp. collection of >3000 accessions initially provided by USDA (Department of Agriculture, Pullman, WA, USA), JIC (John Innes Center, Norwich, UK), CRF (Centro Nacional de Recursos Fitogenéticos, Madrid, Spain), CGN (CPRO-DLO, Wageningen, The Netherland), IPK (Leibniz Institute of Plant Genetics and Crop Plant Research, Gatersleben, Germany) and ICARDA (International Center for Agricultural Research in the Dry Areas, Aleppo, Syria). The collection is representative of the different *Pisum* species and subspecies including accessions from *P*. *sativum*, *P*. *fulvum*, *P*. *abyssinicum* and the subspecies *sativum*, *elatius*, *cinereum*, *jomardii*, *thebaicum* and *transcaucasicum* of *P*. *sativum.* In addition, it comprises cultivated, wild and landrace pea types from worldwide origin, associated with large genetic and morphologic diversity (Figure 1, Table 1 and Supplementary Table S1).

### 4.2. DNA Extraction, Library Construction, and Sequencing

The pea core collection was genotyped with the DArTSeq approach by Diversity Arrays P/L (Canberra, Australia). For this, the third composed leave from 20 seedlings (two weeks old), grown under controlled conditions, for each accession, was harvested, pooled together, flash-frozen in liquid nitrogen and freeze-dried. Then, DNA was extracted following the method stipulated by Diversity Arrays P/L, as previously described [37]. DNA quality was assessed by electrophoresis on 0.8% agarose gel. DNA was quantified by fluorescence at 504 nm_Ex_/531 nm_Em_ on an HT Synergy microplate reader (Biotek, Winooski, VT, USA) with Quantifluor^®^ DsDNA system (Promega Corporation, Madison, WI, USA), following manufacturer recommendations. The DNA samples were then adjusted at 20 ng/µL before DArT marker analysis, using the high-density Pea DArTseq 1.0 array (50,000 markers), adapted for wild *Pisum* spp. accessions, as previously described [37]. Complexity reduction with the *Pst*I and *Mse*I restriction enzymes, library construction, amplification and Illumina sequencing were performed by Diversity Arrays P/L, as described in [37]. DArTSeq sequence analysis retrieved two sets of markers, SNPs and presence–absence sequence variants (SilicoDArT), collectively referred to as DarTSeq markers. Data cleaning was then performed for both DarT markers to remove low-quality and non-polymorphic markers, as described before [21,56]. Accordingly, DArT markers with >20% missing data, MAF < 5% and heterozygosity > 10% were removed from the analysis. In parallel, DArT markers were Blast-mapped onto the *Pisum* reference genome sequences [34] (threshold parameter: E-value = 5 × 10^−4^ and min % identify = 80%). The mapped markers were distributed to each chromosome with the LinkageMapView package in R [57]. Genetic diversity indices, including PIC, Ar, He, Ho and F_IS_, were calculated using the radiator and diveRsity R packages with 1000 bootstraps in R [58,59].

### 4.3. Population Structure of the Pea Core Collection

The population structure of the *Pisum* core collection was inferred with the SilicoDArT dataset after filtering markers in LD with PLINK v1.9 [60]. LD filtering was performed with the pruning method, with a window size of 200 markers and an r^2^ threshold of 0.1, leading to a total of 4000 SilicoDArT markers, of which 2880 were homogeneously distributed onto the seven pea chromosomes (Supplementary Table S2) and 583 mapped to unanchored contigs. Upon LD filtering, population structure was established with STRUCTURE 2.3.4 [61], using the admixture model with correlated allele frequencies between populations, which was shown as the optimum model for fine population structure [62]. Ten independent simulations were performed for each k from k = 1 to k = 15. Each simulation consisted of 10,000 burn-in and 20,000 iterations. Longer burn-in or MCMC did not significantly change the results. The optimal number of k and the percentages of admixture of each accession (Q-matrix) were then given by STRUCTURE HARVESTER [63], according to the Δk method [64]. For subsequent analyses, an accession was assigned to a subpopulation when it had more than 60% membership to this subpopulation. The STUCTURE Q matrix was visualised with the online software STUCTURE PLOT [65]. PCA was also performed with the full *Pisum* SilicoDArT dataset to infer the structure of the *Pisum* collection. PCA was estimated with the function “prcomp” in R verion 4.2.1 [66] and plotted in R with the ggfortify [67] and ggplot2 packages [68] under RStudio version 2022.07.2 build 576 [69].

### 4.4. Phylogenetic Relationship of the Pea Core Collection

The phylogenetic relationship of the 325 pea accessions was inferred using the MEGA X version 2.4 [70] with the full SilicoDArT dataset. For this, a p-distance matrix [71] was estimated from the SilicoDArT matrix after pairwise deletion of the gaps, using the HKY substitution model [72] with gamma distribution, which was the optimal substitution model according to the BIC criterion as estimated with the MEGA X software. Pairwise gap deletion removes all sites with more than 5% alignment gaps, missing data, and ambiguous bases from the marker matrix, leading to a total of 11,635 polymorphic sites. Then, a phylogenetic tree was reconstructed with the NJ method [73] with 1000 bootstrapping replicates based on the p-distance matrix. Upon phylogenetic tree reconstruction, the tree was edited with the MEGA X tree editor to colour each branch according to the STRUCTURE subpopulation.

### 4.5. Linkage Disequilibrium

The disequilibrium matrix summarising pairwise measures of LD was estimated for SilicoDArT and SNP datasets, through a TASSEL 5 software [74] with a sliding window of 100 markers. An LD test was performed for all intrachromosomal marker pairs. To investigate the average LD decay (LD_50_) in the whole genome and per chromosome, significant intra-chromosomal r^2^ values were plotted against the physical distance (Mbp) between markers, with R using the function LDit developed by Ross-Ibarra group (Davis University, USA; https://github.com/rossibarra/r_buffet/blob/master/LDit.r, accessed on 23 June 2022). Average LD_50_ was then estimated in R, as described in Marroni et al. [75].

## Figures and Tables

**Table 1:** Composition of the *Pisum* core collection according to the original passport data from germplasm banks.

Species	Number of Accessions
*P. sativum*	167
*P. sativum* subsp*. sativum*	23
*P. sativum* subsp*. sativum* var. *sativum*	37
*P. sativum* subsp*. sativum* var. *arvense*	32
*P. sativum* subsp. *elatius*	7
*P. sativum* subsp. *elatius* var. *elatius*	24
*P. sativum* subsp. *elatius* var. *pumilio*	3
*P. sativum* subsp. *jomardii*	7
*P. sativum* subsp. *transcaucasicum*	2
*P. sativum* subsp. *thebaicum*	2
*P. sativum* subsp. *cinereum*	1
*P. abyssinicum*	7
*P. fulvum*	13
Total	325

**Table 2:** Genetic diversity indexes of DArTSeq markers.

	SilicoDArT			SNP		
	Mean	Min	Max	Mean	Min	Max
PIC	0.295	0.006	0.499	0.267	0.013	0.594
MAF	0.41	0.052	0.997	0.532	0.046	0.975
Ar	1.783	1	2.91	1.515	0.4	2
Ho	0.06	0	0.7	0.035	0	0.78
He	0.217	0	0.5	0.148	0	0.5
F_IS_	0.692	−0.102	1	0.707	−0.592	1
F_IS_ Low	0.615	−0.675	1	0.588	−0.905	1
F_IS_ High	0.762	−0.058	1.1412	0.815	−0.375	1.305

**Table 3:** Distribution of mapped DArTSeq markers on pea chromosomes.

	Total Lenght (Mbp)	Marker Number		Chromosome Coverage		Mean Distance Between Markers		Marker Density	
		SilicoDarT	SNP	SilicoDarT	SNP	SilicoDarT	SNP	SilicoDarT	SNP
Chr1	372.17	2388	1284	0.04–372.1	0.24–372.0	0.156	0.290	6.4	3.5
Chr2	427.6	2157	1068	0.27–427.4	0.03–427.4	0.198	0.401	5.0	2.5
Chr3	437.56	2331	1194	0.09–437.5	0.09–437.5	0.188	0.367	5.3	2.7
Chr4	446.35	2716	1341	0.03–446.3	0.03–446.3	0.164	0.333	6.1	3.0
Chr5	579.27	3696	1993	0.09–579.1	0.14–579.1	0.157	0.291	6.4	3.4
Chr6	480.42	2970	1672	0.23–480.4	0.21–480.4	0.162	0.287	6.2	3.5
Chr7	491.38	3253	1573	0.05–491.3	0.05–491.1	0.151	0.312	6.6	3.2
Whole genome		19,514	11,511			0.166	0.319	6.0	3.1

**Table 4:** Critical values of LD and LD-decay distance estimated for the DArTSeq markers.

	MeanLD		LD_90_		Dist LD50 (Mbp)	
	SilicoDarT	SNP	SilicoDarT	SNP	SilicoDarT	SNP
Chr1	0.104	0.12	0.244	0.28	0.60	1.58
Chr2	0.091	0.104	0.206	0.239	0.32	0.82
Chr3	0.101	0.114	0.234	0.267	0.60	1.83
Chr4	0.09	0.101	0.203	0.242	0.25	0.69
Chr5	0.104	0.112	0.239	0.264	0.54	1.20
Chr6	0.136	0.159	0.35	0.42	1.05	3.19
Chr7	0.086	0.099	0.191	0.23	0.24	0.78
Whole genome	0.103	0.118	0.236	0.28	0.48	1.38

**Table 5:** Characteristics of STRUCTURE subpopulations.

Clusters	Membership ^a^	Average Dist. ^b^	F_st_
K = 3			
1	28.16	0.2130	0.2268
2	56.83	0.2262	0.3154
3	15.01	0.1514	0.4315
K = 6			
1	14.25	0.1836	0.3316
2	21.08	0.2141	0.3504
3	21.39	0.1670	0.4147
4	21.35	0.1667	0.4688
5	12.57	0.1424	0.4975
6	9.37	0.1305	0.5404

^a^ Percentage of pea accession clustered in the subpopulation. ^b^ Average distance between pea accessions within subpopulation.

## Data Availability

GBS FASTQ raw data generated in this work is available at the NCBI SRA (Bioproject PRJNA890072). Summary and description of the DArTseq markers generated in this work are available in Zenodo, at https://dx.doi.org/10.5281/zenodo.7180467.

## Supplementary Materials

The following supporting information can be downloaded at: https://www.mdpi.com/article/10.3390/ijms24032470/s1.
