# Supplementary material for: Genetic Diversity and Population Structure of a Wide Pisum spp. Core Collection
Source: Int J Mol Sci. 2023 Jan 27;24(3):2470. doi: 10.3390/ijms24032470 (PMC9916889; doi:10.3390/ijms24032470)
Supplement: Supplementary file 1 [file ijms-24-02470-s001.zip › ijms-2148776-supplementary.pdf]

Table S1. Original passport information of the IAS pea core collection

Acc. Nº	Reference	Species	Name	Origen	Mat[1]	PS[2]	Flower Color
1	PI 109865	*P. sativum*	ARVEJAS AMARILLAS	Venezuela	Land.	Adm.	White
2	PI 117910	*P. sativum*	ERVILHA ANA	Brazil	Cult.	Q3	White
3	PI 140297	*P.s. sativum*	No. 6192	Iran	Land.	Adm.	White
4	PI 142442	*P. sativum*	ALBERJON	Peru	Cult.	Adm.	White
5	PI 142774	*P. sativum*	G 1704	Mexico	Land.	Adm.	Purple
6	PI 142776	*P. sativum*	G 1705	Mexico	Land.	Adm.	White
7	PI 142776	*P. sativum*	G 1705	Mexico	Land.	Adm.	White
8	PI 143483	*P. sativum*	No. 7351	Azerbaijan	Land.	Adm.	White
9	PI 143484	*P. sativum*	CPI 135298	Azerbaijan	Land.	Adm.	White
10	PI 143486	*P. sativum*	No. 7790	Iran	Land.	Adm.	White
11	PI 153351	*P. sativum*	ARVEJAS VERDES	Ecuador	Land.	Adm.	White
12	PI 162568	*P. sativum*	ORGULLO DEL MERCADA	Argentina	Cult.	Q3	White
13	PI 162692	*P. sativum*	CUARENTONA	Argentina	Cult.	Adm.	White
14	PI 162693	*P. sativum*	OJO NEGRO	Argentina	Cult.	Adm.	White
15	PI 162693	*P. sativum*	OJO NEGRO	Argentina	Cult.	Adm.	White
16	PI 162910	*P. sativum*	P. NO. 7	Paraguay	Land.	Q3	White
17	PI 164568	*P. sativum*	Patani	India	Land.	Q4	White
18	PI 166082	*P.s. sativum*	Matar	India	Land.	Q6	Purple
19	PI 195405	*P. sativum*	QUEZALTENANGO	Guatemala	Land.	Adm.	Purple
20	PI 203065	*P. sativum*	G 6821	Finland	Land.	Adm.	White
21	PI 204305	*P. sativum*	COLLEGIAN	Australia	Cult.	Adm.	White
22	PI 204667	*P.s. sativum* var *sativum*	STIJFSTRO	Netherland	Cult.	Q3	White
23	PI 220175	*P. sativum*	No. 150	Afganistan	Land.	Q6	Purple
24	PI 220673	*P. sativum*	Moshong	Afganistan	Land.	Adm.	White
25	PI 222069	*P. sativum*	Moshong	Afganistan	Wild	Q6	Purple
26	PI 234262	*P. sativum*	Carlon	USA	Cult.	Adm.	White
27	PI 254625	*P. sativum*	KELLERVA	Finland	Land.	Adm.	White
28	PI 254626	*P. sativum*	LIMA	Australia	Cult.	Adm.	Purple
29	PI 261678	*P. sativum*	Co No. D-237	Netherland	Land.	Adm.	Purple
30	PI 262189	*P. sativum*	BIG PEA	Costa Rica	Land.	Adm.	White
31	PI 266069	*P. sativum*	LINE NO. 110	Sweden	Breed.	Adm.	White
32	PI 269760	*P.s. sativum* var *arvense*	G 16701	UK	Breed.	Adm.	White
33	PI 269763	*P.s. jomardii*	Aa86	UK	Land.	Q2	Purple
34	PI 269786	*P. sativum*	Aa96	UK	Land.	Adm.	Purple
35	PI 272143	*P.s. thebaicum*	THEBAICUM RUBY	Germany	Land.	Adm.	Purple
36	PI 272151	*P. sativum*	UNIFLORUM	Germany	Land.	Adm.	Purple
37	PI 272153	*P. sativum*	HIEMALE	Greece	Land.	Q2	Purple
38	PI 272156	*P. sativum*	HIEMALE	Greece	Land.	Adm.	Purple

Acc. Nº	Reference	Species	Name	Origen	Mat[1]	PS[2]	Flower Color
39	PI 280621	*P. sativum*	AMPLISSIMO SPARTANEC	Rusia	Land.	Q3	White
40	PI 280623	*P. sativum*	AMPLISSIMO PULAVSKIJ	Poland	Land.	Adm.	White
41	PI 306592	*P. sativum*	G 19029	Hungary	Land.	Q2	Purple
42	PI 312136	*P. sativum*	ALBERGA	Guatemala	Land.	Adm.	White
43	PI 319373	*P. sativum*	CHICHARO SERRANO	Mexico	Land.	Adm.	White
44	PI 326194	*P. sativum*	Co No. 22340	Mexico	Land.	Adm.	White
45	PI 343326	*P. sativum*	G 18456	USA	Land.	Q5	White
46	PI 343329	*P. sativum*	G 18459	USA	Land.	Adm.	Purple
47	PI 343935	*P. sativum*	6922	Ethiopia	Land.	Adm.	White
48	PI 343962	*P. sativum*	22662	Turkey	Wild	Adm.	White
49	PI 343965	*P. sativum*	22706	Turkey	Land.	Adm.	White
50	PI 343965	*P. sativum*	22706	Turkey	Land.	Adm.	Purple
51	PI 343969	*P.s. sativum* var *sativum*	ARAKA	Turkey	Land.	Q3	White
52	PI 343981	*P.s. sativum* var *sativum*	22654	Turkey	Land.	Q3	White
53	PI 343984	*P.s. sativum*	22712	Turkey	Land.	Adm.	Purple
54	PI 343993	*P.s. sativum* var *arvense*	22652	Turkey	Land.	Adm.	Purple
55	PI 347282	*P. sativum*	PLP 11	India	Land.	Q5	White
56	PI 347316	*P. sativum*	PLP 68	India	Land.	Q4	White
57	PI 347317	*P. sativum*	PLP 71	India	Land.	Q5	White
58	PI 347319	*P. sativum*	PLP 73	India	Land.	Q4	White
59	PI 347321	*P. sativum*	PLP 88	India	Land.	Q5	White
60	PI 347323	*P. sativum*	PLP 89	India	Land.	Q5	White
61	PI 347326	*P. sativum*	PLP 93	India	Land.	Q5	White
62	PI 347328	*P. sativum*	PLP 99	India	Land.	Adm.	White
63	PI 347330	*P. sativum*	PLP 102	India	Land.	Q5	White
64	PI 347332	*P. sativum*	PLP 104	India	Land.	Q5	White
65	PI 347333	*P. sativum*	PLP 105	India	Land.	Q5	White
66	PI 347334	*P. sativum*	PLP 109	India	Land.	Adm.	White
67	PI 347335	*P. sativum*	PLP 113	India	Land.	Q5	White
68	PI 347336	*P. sativum*	PLP 118	India	Land.	Q5	White
69	PI 347338	*P. sativum*	PLP 126	India	Land.	Adm.	White
70	PI 347342	*P. sativum*	PLP 154	India	Land.	Q4	White
71	PI 347343	*P. sativum*	PLP 156	India	Land.	Adm.	White
72	PI 347347	*P. sativum*	PLP 173	India	Land.	Q5	White
73	PI 347348	*P. sativum*	PLP 182	India	Land.	Q5	White
74	PI 347356	*P. sativum*	PLP 218	India	Land.	Q5	White
75	PI 347357	*P. sativum*	PLP 219	India	Land.	Q5	White
76	PI 347359	*P. sativum*	PLP 222	India	Land.	Q5	White
77	PI 347366	*P. sativum*	PLP 266	India	Land.	Q4	White
78	PI 347367	*P. sativum*	PLP 268	India	Land.	Q5	White

Acc. Nº	Reference	Species	Name	Origen	Mat[1]	PS[2]	Flower Color
79	PI 347370	*P. sativum*	PLP 278	India	Land.	Q5	White
80	PI 347372	*P. sativum*	PLP 297	India	Land.	Q4	White
81	PI 347373	*P. sativum*	PLP 301	India	Land.	Q4	White
82	PI 347374	*P. sativum*	PLP 303	India	Land.	Q4	White
83	PI 347375	*P. sativum*	PLP 304	India	Land.	Q4	White
84	PI 347383	*P. sativum*	PLP 316	India	Land.	Q4	White
85	PI 347385	*P. sativum*	PLP 320	India	Land.	Q5	White
86	PI 347388	*P. sativum*	PLP 330	India	Land.	Q4	White
87	PI 347389	*P. sativum*	PLP 332	India	Land.	Adm.	White
88	PI 347401	*P. sativum*	PLP 363	India	Land.	Adm.	White
89	PI 347471	*P. sativum*	PLP 450	India	Land.	Adm.	White
90	PI 358642	*P.s. sativum* var *arvense*	22793	Ethiopia	Land.	Q3	White
91	PI 379612	*P. sativum*	WEIBULL 700	Sweden	Cult.	Adm.	White
92	PI 385981	*P. sativum*	ONWARD	UK	Cult.	Q3	White
93	PI 399129	*P. sativum*	FLORIDA	Germany	Cult.	Q3	White
94	PI 494079	*P. sativum*	G 27917	Chile	Land.	Adm.	White
95	PI 560065	*P. fulvum*	CPI 134669	Israel	Wild	Q1	Orange
96	PI 560067	*P. fulvum*	CPI 134471	Israel	Wild	Q1	Orange
97	PI 595933	*P. fulvum*	ATC 113	Australia	Wild	Q1	Orange
98	PI 595945	*P. fulvum*	CPI 53306	Jordan	Wild	Q1	Orange
99	PI 595947	*P. fulvum*	VIR 2523	Israel	Wild	Q1	Orange
100	JI 85	*P. sativum*	P. sativum-Afghanistan	Afganistan	Land.	Q6	Pink
101	JI 156	*P. sativum*	P. sativum-USSR	Sudan	Land.	Q2	Pink
102	JI 156	*P. sativum*	P. sativum-USSR	Sudan	Land.	Q2	Pink
103	JI 262	*P.s. elatius* var *elatius*	P. elatius	Turkey	Wild	Q1	Purple
104	JI 263	*P.s. sativum* var *arvense*	P. sativum-Balkans	Greece	Wild	Adm.	Purple
105	JI 228	*P. sativum*	P. sativum-Bolivia	Bolivia	Land.	Adm.	Purple
106	JI 209	*P.s. sativum* var *arvense*	P. sativum arvense	India	Land.	Adm.	Purple
107	JI 209	*P.s. sativum* var *arvense*	P. sativum arvense	India	Land.	Adm.	Purple
108	JI 207	*P.s. sativum* var *sativum*	P. sativum choresmicum	Uzbekistan	Land.	Adm.	White
109	JI 224	*P. fulvum*	P. fulvum	Israel	Wild	Q1	Orange
110	JI 196	*P.s. transcaucasicum*	P. sativum-Georgia	Georgia	Land.	Adm.	Purple
111	JI 190	*P. sativum*	wiraig	Sudan	Land.	Q2	Purple
112	JI 189	*P. sativum*	wiraig	Sudan	Land.	Q2	Purple
113	JI 185	*P. sativum*	wiraig	Sudan	Land.	Q2	Purple
114	JI 267	*P. sativum*	P. sativum-Greece	Greece	Wild	Q2	Purple
115	JI 268	*P. sativum*	P. sativum-Crete	Crete	Wild	Adm.	Purple
116	JI 275	*P. sativum*	P. sativum-Crete	Crete	Wild	Adm.	Purple
117	JI 280	*P. sativum*	P. sativum-Albania	Albania	Wild	Adm.	Purple

Acc. Nº	Reference	Species	Name	Origen	Mat[1]	PS[2]	Flower Color
118	JI 288	*P. sativum*	P. sativum-Greece	Greece	Wild	Adm.	Purple
119	JI 502	*P. sativum*	Rondo	Netherland	Cult.	Q3	White
120	JI 701	*P. sativum*	P. sativum-Italy	Italy	Land.	Adm.	Purple
121	JI 1030	*P. sativum*	P. sativum-Iran	Iran	Land.	Adm.	Purple
122	JI 1057	*P. sativum*	ANTIOQUIA I CHILENA	Colombia	Land.	Q3	White
123	JI 1089	*P.s. sativum* var *arvense*	P. elatius	Turkey	Land.	Adm.	Purple
124	JI 1107	*P. sativum*	keerau pea	Nepal	Land.	Q6	Purple
125	JI 1213	*P. sativum*	erylis	France	Cult.	Q3	White
126	JI 1345	*P. sativum*	P. sativum-Mongolia	Mongolia	Land.	Adm.	White
127	JI 1346	*P. sativum*	P. sativum-Mongolia	Mongolia	Land.	Q6	Purple
128	JI 2263	*P. sativum*	WILD TUNESIAN	Germany	Land.	Adm.	Pink
129	JI 2265	*P. sativum*	P. sativum var. hiemale	Albania	Land.	Adm.	Purple
130	JI 2356	*P. sativum*	P. sativum-Nepal	Nepal	Land.	Adm.	Purple
131	JI 2385	*P. abyssinicum*	Pisum sp.-Yemen	Yemen	Land.	Q1	Lilac
132	JI 2387	*P. sativum*	P. sativum-Ethiopia	Ethiopia	Land.	Adm.	Purple
133	JI 2545	*P. sativum*	P. sativum-Pakistan	Pakistan	Land.	Q6	Purple
134	BGE001004	*P.s. sativum*	Garvanzo enano	Spain	Land.	Adm.	White
135	BGE001034	*P.s. sativum* var *sativum*	Pesol	Spain	Land.	Q3	White
136	BGE001121	*P.s. sativum* var *sativum*	Negrer	Spain	Land.	Q3	White
137	BGE001121	*P.s. sativum* var *sativum*	Negrer	Spain	Land.	Q3	White
138	BGE001662	*P.s. sativum*	Chicharo	Spain	Land.	Adm.	Purple
139	BGE002168	*P.s. sativum*	Tito	Spain	Land.	Adm.	Purple
140	BGE002168	*P.s. sativum*	Tito	Spain	Land.	Adm.	White
141	BGE003315	*P.s. sativum*	Tirabeque	Spain	Land.	Adm.	Purple
142	BGE004710	*P.s. sativum*	Ervilha	Portugal	Land.	Adm.	Purple
143	BGE004713	*P. sativum* spp. *sativum*	Ervilha	Portugal	Land.	Adm.	Purple
144	BGE004958	*P. sativum* spp. *sativum*	Ervilhoto	Portugal	Land.	Adm.	Purple
145	BGE006125	*P. sativum* spp. *sativum*	Grizeu farroba	Portugal	Land.	Adm.	Purple
146	BGE006126	*P.s. sativum* var *sativum*	Ervilha	Portugal	Land.	Adm.	White
147	BGE019594	*P. sativum* spp. *sativum*	Arveja	Spain	Land.	Adm.	White
148	BGE022159	*P.s. sativum* var *arvense*	Bisalto	Spain	Land.	Adm.	Purple
149	BGE020326	*P.s. sativum* var *arvense*	Bisalto del terreno	Spain	Land.	Adm.	Purple
150	BGE023256	*P.s. sativum*	Guisante	Spain	Land.	Adm.	White
151	BGE025263	*P.s. sativum* var *sativum*	Guisante verde	Spain	Land.	Adm.	White
152	BGE025267	*P.s. sativum*	Mangano;Presol;Guisant e claro	Spain	Land.	Adm.	White
153	BGE025270	*P.s. sativum*	Guisante negro	Spain	Land.	Adm.	Purple
154	BGE026428	*P.s. sativum* var *sativum*	Guisante rastrero	Spain	Land.	Q3	White

Acc. Nº	Reference	Species	Name	Origen	Mat[1]	PS[2]	Flower Color
155	BGE026429	*P.s. sativum*	Arvilla	Spain	Land.	Adm.	Purple
156	CGN16690	*P. sativum*		Italy	Land.	Adm.	Purple
157	CGN03277	*P. sativum*	NPE 378	Pakistan	Land.	Q6	Purple
158	CGN13253	*P. sativum*	P. sativum-Ethiopia	Ethiopia	Land.	Adm.	Purple
159	CGN16640	*P. sativum*	Khadraa	Sudan	Land.	Adm.	White
160	CGN16562	*P. sativum*	JI 1543	Mongolia	Wild	Adm.	Purple
161	CGN16571	*P.s. jomardii*	P. jomardii	Egypt	Wild	Q2	Pink
162	CGN16581	*P. sativum*	JI 93	Afganistan	Land.	Adm.	Brick Red
163	CGN16639	*P. sativum*	JI 171	Ethiopia	Land.	Adm.	Purple
164	CGN16679	*P.s. cinereum*	JI 204	Russia	Wild	Adm.	Purple
165	CGN16582	*P. sativum*	Keerau pea	Nepal	Land.	Q6	Purple
166	CGN16684	*P. sativum*		Greece	Land.	Adm.	Purple
167	CGN16646	*P. sativum*		Mongolia	Land.	Adm.	Purple
168	CGN16636	*P. abyssinicum*	P. abyssinicum	Ethiopia	Land.	Q1	Lilac
169	CGN03328	*P. sativum*	NPE 1210.362A	Pakistan	Land.	Q6	Purple
170	CGN03170	*P. sativum*	Turkey-19	Irak	Land.	Q4	White
171	CGN03190	*P. sativum*	Kulur	Turkey	Land.	Adm.	Purple
172	CGN03245	*P. sativum*	Ethiopia-32	Ethiopia	Land.	Adm.	Purple
173	CGN03165	*P. sativum*	Turkey-16	Turkey	Land.	Adm.	Purple
174	CGN03289	*P. sativum*	NPE 1175.346	Pakistan	Land.	Q6	Purple
175	CGN03171	*P. sativum*	Selection 266/1	Turkey	Breed.	Adm.	White
176	CGN03290	*P. sativum*	NPE 1180.392	Pakistan	Land.	Q6	Purple
177	CGN03305	*P. sativum*	NPE 1169.248	Pakistan	Land.	Q6	Purple
178	CGN02921	*P.s. sativum* var *sativum*	Semi Nano Ideal	Italy	Cult.	Q3	White
179	CGN03003	*P.s. sativum* var *sativum*	Petit Provencal	France	Cult.	Q3	White
180	CGN03273	*P. sativum*	950 3e	Peru	Breed.	Adm.	Purple
181	CGN03229	*P. sativum*	Ethiopia-31	Ethiopia	Land.	Q4	Purple
182	PI 413686	*P. sativum*	FELICITAS	Hungary	Cult.	Adm.	Purple
183	PI 477371	*P. sativum*	ROSAKRONE	Denmark	Cult.	Adm.	Pink
184	PI 307666	*P. sativum*	VERJA	Costa Rica	Land.	Adm.	White
185	PI 307666	*P. sativum*	VERJA	Costa Rica	Land.	Adm.	Purple
186	PI 324693	*P. sativum*	ABESINIJAS	Hungary	Cult.	Q3	White
187	PI 324705	*P. sativum*	No. 830	France	Un.	Adm.	Purple
188	PI 355905	*P.s. sativum* var *sativum*	KAIRYO AOTENASHI	Japan	Cult.	Q3	White
189	PI 241593	*P. sativum*	G 6571	Taiwan, China	Un.	Adm.	Purple
190	PI 273207	*P.s. elatius*	9006/60	Bulgaria	Land.	Q1	Purple
191	PI 266070	*P.s. sativum* var *sativum*	LINE NO. 930	Sweden	Breed.	Adm.	White
192	PI 198074	*P. sativum*	GORSDAGSART III	Sweden	Land.	Adm.	White
193	PI 357292	*P.s. sativum* var *sativum*	KIFLICA	North Macedonia	Cult.	Q3	White

Acc. Nº	Reference	Species	Name	Origen	Mat[1]	PS[2]	Flower Color
194	PI 357293	*P.s. sativum* var *sativum*	DEBARSKI	North Macedonia	Cult.	Q3	White
195	PI 249645	*P. sativum*	B.R. 178	India	Land.	Adm.	Purple
196	PI 357048	*P.s. elatius*	PLP 514	India	Un.	Q6	Purple
197	PI 357289	*P.s. sativum* var *sativum*	RAN	North Macedonia	Cult.	Q3	White
198	PI 253968	*P.s. elatius*	Co No. K1722	Afganistan	Land.	Q6	Purple
199	PI 103058	*P. sativum*	No. 10	China	Cult.	Adm.	White
200	PI 180329	*P. sativum*	Watana	India	Land.	Q2	Purple
201	PI 184131	*P.s. sativum* var *sativum*	No. 310	Serbia	Land.	Q3	White
202	PI 124478	*P. sativum*	Matar	Pakistan	Land.	Adm.	Purple
203	PI 124479	*P. sativum*	Matar	Pakistan	Land.	Adm.	White
204	PI 124479	*P. sativum*	Matar	Pakistan	Land.	Adm.	Purple
205	JI 2480	*P. sativum*	CGN 3352	Peru	Breed.	Adm.	White
206	JI 1951	*P. sativum*	P. sativum-China	China	Cult.	Adm.	White
207	JI 2302	*P.s. sativum* var *sativum*	B76-197 (STRATAGEM)	Sweden	Breed.	Q3	White
208	JI 1566	*P. sativum*	Almota	USA	Cult.	Q3	White
209	PI 608038	*P. sativum*	74SN5	USA	Cult.	Adm.	White
210	PI 613100	*P. sativum*	MINI	USA	Cult.	Adm.	White
211	Atc-4235-53	*P. sativum*	Atc-4235-53	Australia	Breed.	Adm.	White
212	Boreen	*P. sativum*	Boreen	Australia	Cult.	Q3	White
213	Danclale	*P. sativum*	Danclale	Australia	Cult.	Adm.	Purple
214	Kagpa	*P. sativum*	Kagpa	Australia	Cult.	Adm.	Pink
215	M5	*P. sativum*	M5	Australia	Cult.	Q3	Purple
216	Pinochio	*P. sativum*	Pinochio	Denmark	Cult.	Q3	White
217	B 99-114	*P. sativum*	B 99-114	Czech Republic	Breed.	Q3	White
218	AGT 205,21	*P. sativum*	AGT 205,21	Czech Republic	Breed.	Q3	White
219	Morris	*P. sativum*	Morris	Czech Republic	Cult.	Q3	White
220	JI 1210	*P. sativum*	erygel	France	Cult.	Q3	White
221	JI 1412	*P. sativum*	Marlin	USA	Cult.	Q3	White
222	JI 1559	*P. sativum*	Mexique 4	Mexico	Cult.	Adm.	White
223	JI 1747	*P. sativum*	Almires	Germany	Cult.	Q3	White
224	JI 1760	*P.s. sativum* var *sativum*	Consort-af	UK	Cult.	Q3	White
225	JI 210	*P. sativum*	Lucknow Boniya	India	Cult.	Adm.	White
226	JI 252	*P. sativum*	P. sativum-Ethiopia	Ethiopia	Land.	Q6	Purple
227	JI 82	*P. sativum*	P. sativum-Afghanistan	Afganistan	Land.	Adm.	White
228	Messire	*P.s. sativum* var *sativum*	Messire	France	Cult.	Q3	White
229	Radley	*P.s. sativum* var *sativum*	Radley	UK	Cult.	Q3	White
230	Ballet	*P.s. sativum* var *sativum*	Ballet	UK	Cult.	Q3	White
231	W6 17515	*P.s. sativum* var *sativum*	LITTLE MARVEL	USA	Cult.	Q3	White

Acc. Nº	Reference	Species	Name	Origen	Mat[1]	PS[2]	Flower Color
232	W6 17516	*P.s. sativum* var *sativum*	DARK SKIN PERFECTION	USA	Cult.	Q3	White
233	W6 17517	*P.s. sativum* var *sativum*	NEW ERA	USA	Cult.	Q3	White
234	W6 17518	*P.s. sativum* var *sativum*	NEW SEASON	USA	Cult.	Q3	White
235	W6 17520	*P.s. sativum* var *sativum*	WSU 28	USA	Cult.	Q3	White
236	KEBBY	*P.s. sativum* var *sativum*	Kebby	UK	Cult.	Q3	White
237	POLAR	*P.s. sativum* var *sativum*	Polar	Spain	Cult.	Q3	White
238	W6 17519	*P.s. sativum* var *sativum*	WSU 23		Cult.	Adm.	White
239	W6 17521	*P.s. sativum* var *sativum*	WSU 31		Cult.	Q3	White
240	BGE023667	*P.s. sativum*	Guisante	Spain	Land.	Adm.	White
241	BGE025727	*P.s. sativum*	Guisante	Spain	Land.	Adm.	Purple
242	PI 358608	*P.s. sativum* var *arvense*	22770B	Ethiopia	Land.	Q4	Purple
243	PI 358609	*P. abyssinicum*	WAT	Ethiopia	Wild	Q1	Pink
244	PI 173055	*P.s. elatius* var *elatius*	HATUN BAKLERI	Turkey	Land.	Adm.	Lilac
245	PI 120617	*P.s. elatius* var *elatius*	No. 738	Turkey	Land.	Adm.	Purple
246	PI 273209	*P.s. elatius* var *elatius*	9009/60	Russia	Land.	Adm.	Purple
247	PI 344003	*P.s. elatius* var *elatius*	22703	Turkey	Wild	Adm.	Purple
248	PI 344005	*P.s. elatius* var *elatius*	22611	Greece	Wild	Q1	Purple
249	PI 344006	*P.s. elatius* var *elatius*	22618	Greece	Wild	Q1	Purple
250	PI 343976	*P.s. elatius* var *elatius*	22716	Turkey	Wild	Q1	Purple
251	PI 505059	*P.s. elatius* var *elatius*	ILCA 5076	Sudan	Land.	Q2	Pink
252	PI 344010	*P.s. elatius* var *elatius*	22732	Greece	Wild	Q1	Purple
253	PI 344011	*P.s. elatius* var *elatius*	22733	Greece	Wild	Q1	Purple
254	PI 344013	*P.s. elatius* var *elatius*	22735	Greece	Wild	Q1	Purple
255	PI 116056	*P.s. sativum*	Matar	India	Land.	Adm.	Purple
256	PI 505127	*P.s. sativum*	ILCA 5094	Albania	Land.	Adm.	Purple
257	PI 242027	*P.s. jomardii*	G 11764	Denmark	Un.	Q2	Pink
258	PI 269762	*P.s. jomardii*	Aa38	UK	Land.	Q2	Purple
259	PI 343987	*P.s. sativum* var *sativum*	22718	Turkey	Land.	Adm.	White
260	PI 505080	*P.s. sativum*	ILCA 5039	Cyprus	Un.	Adm.	White
261	PI 505111	*P.s. sativum*	ILCA 5075	Syria	Land.	Adm.	Purple
262	PI 268480	*P.s. elatius* var *pumilio*	Co No. 317	Afganistan	Land.	Q6	Purple
263	JI 45	*P.s. transcaucasicum*	P. transcaucasicum	Georgia	Wild	Adm.	Purple
264	JI 198	*P.s. elatius* var *elatius*	P. elatius	Israel	Wild	Adm.	Purple
265	JI 199	*P.s. elatius* var *elatius*	P. elatius	Israel	Wild	Adm.	Purple

Acc. Nº	Reference	Species	Name	Origen	Mat[1]	PS[2]	Flower Color
266	JI 225	*P. abyssinicum*	P. abyssinicum	Ethiopia	Land.	Q1	Lilac
267	JI 227	*P. abyssinicum*	P. abyssinicum	Ethiopia	Land.	Q1	Purple
268	JI 241	*P.s. elatius* var *pumilio*	P. humile	Israel	Wild	Q6	Purple
269	JI 254	*P.s. elatius* var *elatius*	P. elatius	Ethiopia	Wild	Q1	Purple
270	JI 804	*P.s. sativum* var *sativum*	P. tibetanicum		Land.	Adm.	Purple
271	JI 1398	*P. sativum*	P. sativum	China	Land.	Adm.	White
272	JI 1428	*P.s. sativum* var *sativum*	P. tibetanicum	Tibet	Wild	Adm.	Purple
273	JI 1854	*P.s. elatius* var *pumilio*	P. Humile	Israel	Land.	Q6	Purple
274	JI 2116	*P.s. sativum*	P. speciosum	Spain	Land.	Adm.	Purple
275	JI 2202	*P. abyssinicum*	P. Abyssinicum	Yemen	Land.	Q1	Lilac
276	PIS 1318/91	*P.s. elatius*			Un.	Adm.	Lilac
277	CGN10205	*P.s. elatius* var *elatius*	1140175	Turkey	Land.	Adm.	Purple
278	CGN10206	*P.s. elatius* var *elatius*	1145176		Breed.	Adm.	White
279	CGN10193	*P.s. sativum* var *arvense*			Un.	Adm.	Purple
280	IFPI 3365	*P.s. elatius* var *elatius*	IG 52524	Turkey	Wild	Adm.	Purple
281	IFPI 3370	*P.s. elatius* var *elatius*	IG 52529	Turkey	Wild	Adm.	White
282	IFPI 387	*P.s. thebaicum*	IG 49546	USSR	Wild	Adm.	Pink
283	IFPI 436	*P.s. jomardii*	IG 49595	Egypt	Land.	Q2	Purple
284	IFPI 2348	*P.s. sativum* var *arvense*	IG 51507	Ethiopia	Land.	Q4	Purple
285	IFPI 2350	*P.s. sativum* var *arvense*	IG 51509	Ethiopia	Land.	Q4	Purple
286	IFPI 2351	*P.s. sativum* var *arvense*	IG 51510	Ethiopia	Land.	Adm.	White
287	IFPI 2352	*P.s. sativum* var *arvense*	IG 51511	Ethiopia	Land.	Q4	Purple
288	IFPI 2353	*P.s. sativum* var *arvense*	IG 51512	Ethiopia	Land.	Adm.	Purple
289	IFPI 2354	*P.s. sativum* var *arvense*	IG 51513	Ethiopia	Land.	Q4	Purple
290	IFPI 2356	*P.s. sativum* var *arvense*	IG 51515	Ethiopia	Land.	Q4	White
291	IFPI 2357	*P.s. sativum* var *arvense*	IG 51516	Ethiopia	Land.	Q4	Purple
292	IFPI 2358	*P.s. sativum* var *arvense*	IG 51517	Ethiopia	Land.	Adm.	White
293	IFPI 2360	*P.s. sativum* var *arvense*	IG 51519	Ethiopia	Land.	Q4	Purple
294	IFPI 2362	*P.s. sativum* var *arvense*	IG 51521	Ethiopia	Land.	Q4	Purple
295	IFPI 2363	*P.s. sativum* var *arvense*	IG 51522	Ethiopia	Land.	Adm.	Purple
296	IFPI 2364	*P.s. sativum* var *arvense*	IG 51523	Ethiopia	Land.	Q4	Purple
297	IFPI 2365	*P.s. sativum* var *arvense*	IG 51524	Ethiopia	Land.	Q4	Purple
298	IFPI 2367	*P.s. sativum* var *arvense*	IG 51526	Ethiopia	Land.	Adm.	Purple

Acc. Nº	Reference	Species	Name	Origen	Mat[1]	PS[2]	Flower Color
299	IFPI 2369	*P.s. sativum* var *arvense*	IG 51528	Ethiopia	Land.	Adm.	Purple
300	IFPI 2370	*P.s. sativum* var *arvense*	IG 51529	Ethiopia	Land.	Adm.	Purple
301	IFPI 2371	*P.s. sativum* var *arvense*	IG 51530	Ethiopia	Land.	Adm.	White
302	IFPI 2372	*P.s. sativum* var *arvense*	IG 51531	Ethiopia	Land.	Q4	Purple
303	IFPI 2441	*P.s. jomardii*	IG 51600	Denmark	Un.	Q2	Pink
304	IFPI 2495	*P.s. jomardii*	IG 51654	UK	Land.	Q2	Pink
305	IFPI 3232	*P. fulvum*	IG 52391	Syria	Wild	Q1	Orange
306	IFPI 3250	*P. sativum*	IG 52409	Syria	Wild	Adm.	Purple
307	IFPI 3252	*P.s. elatius*	IG 52411	Syria	Wild	Q1	Purple
308	IFPI 3253	*P. fulvum*	IG 52412	Syria	Wild	Q1	Orange
309	IFPI 3257	*P. fulvum*	IG 52416	Syria	Wild	Q1	Orange
310	IFPI 3260	*P. fulvum*	IG 52419	Syria	Wild	Q1	Orange
311	IFPI 3261	*P. fulvum*	IG 52420	Syria	Wild	Q1	Orange
312	IFPI 3262	*P. fulvum*	IG 52421	Syria	Wild	Q1	Orange
313	IFPI 3280	*P.s. elatius*	IG 52439	Syria	Wild	Q1	Purple
314	IFPI 3282	*P.s. elatius*	IG 52441	Syria	Wild	Q1	Purple
315	IFPI 3330	*P.s. elatius* var *elatius*	IG 52489	Turkey	Wild	Q2	Pink
316	IFPI 3334	*P.s. elatius* var *elatius*	IG 52493	Turkey	Wild	Q1	Purple
317	IFPI 3338	*P.s. elatius* var *elatius*	IG 52497	Turkey	Wild	Q1	Purple
318	IFPI 3358	*P.s. elatius* var *elatius*	IG 52517	Turkey	Wild	Adm.	Orange
319		*P.s. elatius* var *elatius*			Wild	Adm.	Purple
320	JI 1006	*P. fulvum*	WBH 2142	Israel	Wild	Q1	Orange
321	Cartouche	*P.s. sativum* var *sativum*	Cartouche		Cult.	Adm.	White
325	PI 273605	*P. sativum*	G 11058	Ecuador	Wild	Adm.	Pink
326	PI 505092	*P.s. sativum* var *arvense*	ILCA 5052	Cyprus	Land.	Adm.	White
327	IFPI 2355	*P.s. sativum* var *arvense*	IG 51514	Ethiopia	Un.	Q4	White
328	JI 130	*P. abyssinicum*	P. abyssinicum	Ethiopia	Land.	Q1	Lilac

[1] Type of Material (Mat.): Landraces (Land.), Cultivar (Cult.), Breeding material (Breed.), Wild or Unknown (Un.).

[2] Population Structure as determined by STRUCTURE for K = 6. Accessions were assigned to a given Q when its percentage of membership was > 60%.

Table S2. Distribution of Silico-DArT markers onto pea genome after LD pruned

	Chromosome lenght (Mbp)	Marker number	Chromosome coverage	Mean distance between markers	Marker density
Chr1	372.17	307	0.04-372.08	1.216	0.8
Chr2	427.6	346	0.03 - 472.40	1.239	0.8
Chr3	437.56	325	6.89-436.8	1.327	0.7
Chr4	446.35	441	0.03 - 446.3	1.014	1.0
Chr5	579.27	471	0.14 - 579.1	1.232	0.8
Chr6	480.42	439	0.26 - 479.7	1.095	0.9
Chr7	491.38	551	0.05 - 491.2	0.892	1.1
Whole genome		2880			0.9

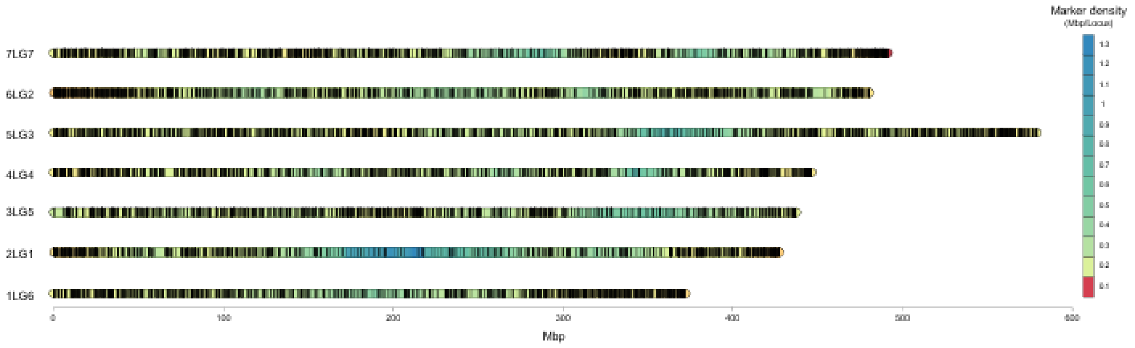

Figure S1. Silico-DArT marker distribution over each pea chromosomes

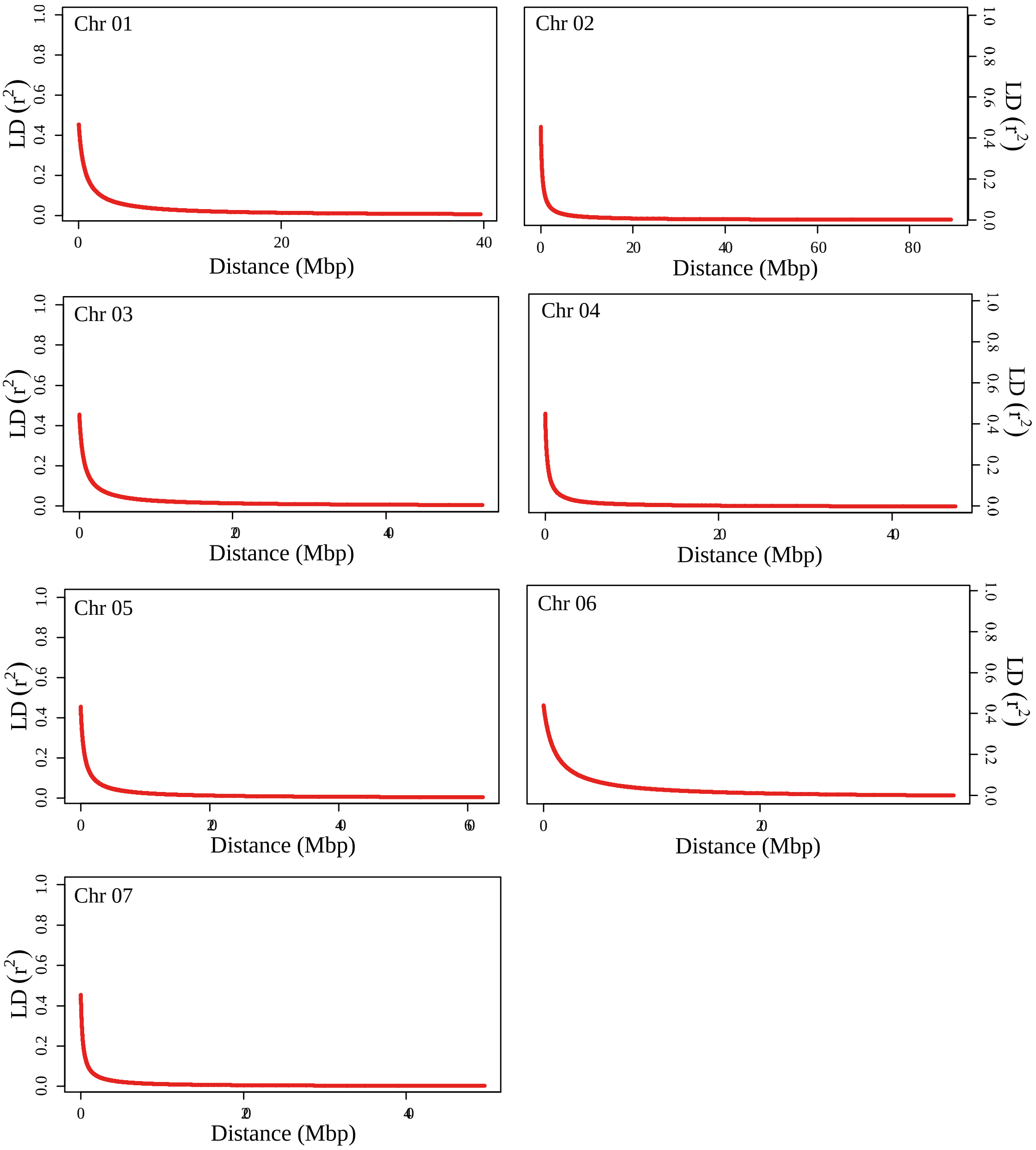

Figure S2. Estimation of the LD decay distance per chromosome
